# Psychedelic exposure in pregnancy: a scoping review to inform perinatal drug safety and clinical counseling

**DOI:** 10.1177/20420986261436104

**Published:** 2026-03-31

**Authors:** Ovie Martin Albert, Alexander Arthur

**Affiliations:** Department of Family Medicine, Cumming School of Medicine, University of Calgary, Calgary, AB, Canada; VODP, Addiction and Mental Health, Centennial Centre for Mental Health and Brain Injury, Alberta Health Services/Recovery Alberta, Ponoka, Alberta, Canada; Department of Family Medicine, University of Saskatchewan, Saskatoon, SK, Canada; St. Joseph’s Hospital, Estevan, SK, Canada

**Keywords:** congenital anomalies, hallucinogens, lysergic acid diethylamide, 3,4-methylenedioxymethamphetamine, pregnancy, prenatal exposure

## Abstract

Psychedelic and psychedelic-adjacent substances, including 3,4-methylenedioxymethamphetamine (MDMA) and classic serotonergic hallucinogens, are undergoing renewed therapeutic investigation and remain in non-medical use. Inadvertent exposure during early, unrecognized pregnancy is clinically plausible, yet pregnancy-specific safety evidence is limited. To map and synthesize the extent, characteristics, and limitations of primary human evidence on prenatal exposure to MDMA, psilocybin, and classic hallucinogens (lysergic acid diethylamide (LSD), mescaline/peyote, and *N,N*-dimethyltryptamine (DMT)/ayahuasca), and to identify clinically relevant evidence gaps for perinatal counseling and pharmacovigilance. Peer-reviewed primary human studies (cohort, case–control, cross-sectional, case series, case reports, and brief reports) describing prenatal exposure with reported maternal, obstetric, neonatal, congenital anomaly, or child neurodevelopmental outcomes were included. Animal and preconception-only studies were excluded. MEDLINE, Embase, PsycINFO, CINAHL, and the Cochrane Library were searched from inception to March 2025. Supplementary methods included Google Scholar screening and citation tracking. Data were charted in duplicate using a standardized form and synthesized descriptively by substance and outcome domain. Consistent with scoping methodology, no formal risk-of-bias assessment or meta-analysis was undertaken. Twenty-three primary human sources (1968–2020) met inclusion criteria: MDMA (*n* = 11), LSD (*n* = 11), and mescaline/peyote (*n* = 1). No eligible primary human pregnancy outcome studies were identified for psilocybin or DMT/ayahuasca. The evidence base was heterogeneous and predominantly comprised small cohorts, teratology service follow-up reports, and case-based publications, frequently limited by self-reported exposure, polysubstance confounding, and inconsistent outcome definitions. Human evidence on prenatal psychedelic exposure remains sparse and methodologically constrained. Absence of data for several substances should not be interpreted as evidence of safety. Clinicians should counsel with explicit acknowledgment of uncertainty while supporting harm reduction and appropriate follow-up. Structured perinatal pharmacovigilance and ethically designed evidence-generation strategies are needed as therapeutic psychedelic research expands.

## Plain language summary

What we know about psychedelic drug exposure during pregnancy

Some people may be exposed to psychedelic drugs such as MDMA (“ecstasy”), LSD, or psilocybin during pregnancy, often before they realize they are pregnant. As interest in psychedelic therapies grows, clinicians are increasingly asked about possible risks to pregnancy and child development. In this review, we examined all available human studies that reported pregnancy or child outcomes following exposure to psychedelic or psychedelic-related substances. We found that the human evidence base is very limited and mostly consists of small observational studies, case reports, and follow-up data from teratology information services. Most available studies focus on MDMA or LSD, while there is little to no direct human pregnancy outcome data for substances such as psilocybin or ayahuasca. Because the available evidence is sparse and highly variable, it is not possible to reliably estimate the risks of miscarriage, birth defects, or long-term child development following psychedelic exposure during pregnancy. This review highlights important gaps in current knowledge and explains why careful, individualized clinical counselling is needed when such exposures occur. The findings are relevant for clinicians, patients, and researchers involved in drug safety and pregnancy care.

## Introduction

### Background and clinical context

Psychedelic and psychedelic-adjacent substances have re-emerged as prominent targets of clinical investigation and public discourse. 3,4-Methylenedioxymethamphetamine (MDMA)-assisted psychotherapy has been evaluated in phase III trials for post-traumatic stress disorder, occurring within an evolving regulatory landscape that has not yet resulted in routine clinical implementation.^1,2,4^ In parallel, therapeutic research programs involving serotonergic psychedelics (e.g., psilocybin) and related agents continue to expand, supported by synthesis of emerging randomized evidence across psychiatric indications.^3^ These developments occur against a backdrop of ongoing non-medical use and variable drug supply characteristics, creating a plausible and increasingly relevant scenario for inadvertent exposure during pregnancy—particularly during early, unrecognized pregnancy—encountered by clinicians in obstetrics, primary care, emergency medicine, and addiction medicine.^3,7–9^

### The pregnancy evidence gap in drug safety

Despite the expanding therapeutic and non-medical landscape, pregnancy-specific safety evidence for many drugs remains limited because pregnant and breastfeeding individuals are commonly excluded from clinical trials. International and national guidance documents have emphasized that routine exclusion has contributed to persistent evidence gaps, leaving clinicians to rely on post-marketing observational data, case reports, and teratology information services for risk counseling.^4–6^ This challenge is especially salient for psychoactive substances, where pregnancy exposure may be under-reported and where confounding by polysubstance use, socioeconomic determinants, and comorbid psychiatric illness complicates causal inference.

Importantly, these limitations do not arise randomly but reflect how pregnancy drug safety evidence is typically generated in domains characterized by therapeutic uncertainty and social stigma. Routine exclusion of pregnant individuals from interventional trials shifts evidence generation into post-marketing and observational contexts, where exposure is often ascertained through self-report or teratology information services rather than a prospective protocolized study. As a result, data tend to accumulate through fragmented case reports, small follow-up cohorts, and heterogeneous documentation practices rather than coordinated surveillance systems. For psychoactive substances in particular, under-reporting, polysubstance co-use, and variable drug purity further shape the character of the available evidence. The observed fragmentation in prenatal psychedelic exposure research, therefore, reflects a structurally constrained evidence environment rather than a series of isolated methodological shortcomings.

Teratology information services provide an important bridge between limited primary evidence and clinical counseling needs. However, their summaries generally reflect the constraints of the underlying literature: small samples, reliance on self-report, uncertain dose and timing, and heterogeneous outcome definitions.^7–9^ In this context, clinicians frequently face counseling questions about fetal risk with limited ability to offer evidence-based quantification of miscarriage risk, congenital anomaly patterns, or longer-term neurodevelopmental outcomes.

### What is known from published human evidence

Among the substances of interest, the most substantive primary human pregnancy evidence pertains to MDMA/ecstasy exposure. Signals and outcomes have been reported through teratology service follow-up and case series, including congenital anomaly reports after prenatal ecstasy exposure.^10–12^ Prospective cohort work has also reported early neurobehavioral findings and subsequent developmental outcomes in infants and children with prenatal MDMA exposure, though interpretation is constrained by frequent co-exposures and potential exposure misclassification.^13–16^ In addition to infant/child outcomes, several pregnancy-specific publications from the same broader research stream contribute clinically relevant information on exposure patterns, maternal characteristics, and maternal outcomes (including postpartum mental health and neurohormonal correlates) that can inform counseling and bias assessment even when infant outcomes are not the primary endpoint.^17–20^

For classic hallucinogens such as lysergic acid diethylamide (LSD), the human literature is older and largely composed of observational reports, brief reports/letters, and case-based publications. Observational follow-up has described pregnancy outcomes following medically administered or reported LSD exposure,^21^ and additional publications have reported reproductive outcomes among LSD users, including congenital anomaly reporting and pregnancy loss patterns, though causality is difficult to determine given limitations in exposure ascertainment and co-exposures.^22^ Reported congenital and ocular malformations in case reports further illustrate the heterogeneity of outcomes and the vulnerability of the evidence base to reporting bias.^23,24^ Other brief reports have discussed teratogenic signals and child outcomes following prenatal exposure, often in the setting of polysubstance use, again limiting inference.^25–28^ A small number of more recent reports continue to appear, including case-based overdose presentations that involve early pregnancy exposure.^29^

For mescaline/peyote, the published primary human pregnancy literature appears extremely limited, with isolated case-based reporting.^30^ For psilocybin, accessible teratology resources emphasize that human pregnancy data are sparse, limiting assessment of risks for miscarriage, congenital anomalies, or pregnancy complications and leaving clinicians without robust evidence for counseling.^9^ Taken together, the existing human literature suggests that the evidence base is likely to remain fragmented across substances, study designs, and outcome domains, with a strong concentration in MDMA and LSD reports and minimal primary reporting for other classic psychedelics.^7–9,30^

Viewed collectively, these findings suggest clinically plausible maternal–fetal pathways that warrant focused attention, even in the absence of high-certainty risk estimates. Prospective MDMA cohorts describing early neurobehavioral differences and persistent motor or developmental signals raise the possibility of altered in utero neurodevelopmental trajectories, particularly given serotonergic modulation during critical windows of fetal brain development.^13–16^ Reported congenital and ocular anomalies in case-based LSD literature, although heterogeneous and methodologically limited, further underscore the need to consider potential developmental vulnerability during early gestation.^23–27,35,36^ In addition, pregnancy-specific publications describing maternal psychiatric profiles and neurohormonal correlates in MDMA-exposed cohorts suggest that maternal physiological and psychological states may represent intermediary pathways through which fetal development could be indirectly affected.^17–20^ While these signals do not establish causality and remain vulnerable to confounding and exposure misclassification, they converge on biologically plausible maternal–fetal pathways involving serotonergic modulation during critical periods of fetal neurodevelopment, potential early gestational vulnerability to congenital anomalies, and indirect effects mediated through maternal physiological and psychiatric states. For obstetric and addiction medicine clinicians, this convergence defines the clinical stakes: prenatal psychedelic exposure cannot be treated as a neutral absence-of-data problem, but rather as a question involving plausible developmental vulnerability under conditions of evidentiary uncertainty.

Importantly, MDMA is pharmacologically distinct from classic serotonergic psychedelics such as LSD and psilocybin, acting primarily through monoaminergic release mechanisms rather than direct 5-HT2A receptor agonism. Findings from MDMA cohorts should therefore not be extrapolated to classic psychedelics, and conversely, the absence of eligible pregnancy outcome data for psilocybin or *N,N*-dimethyltryptamine (DMT)/ayahuasca must not be interpreted as evidence of safety.

### Rationale for a scoping review

A conventional systematic review or meta-analysis is most informative when a sufficiently coherent body of studies exists (e.g., comparable exposure definitions, outcome measurement, and design), enabling quantitative synthesis and effect estimation. In contrast, the human pregnancy literature for psychedelics is anticipated to be sparse, methodologically heterogeneous, and subject to substantial threats to validity (including polysubstance confounding and uncertain timing/dose). Under these conditions, a scoping review is the most appropriate evidence synthesis approach to map what evidence exists, characterize how exposures and outcomes have been studied, and identify knowledge gaps while avoiding over-interpretation of low-certainty signals.^31–34^ In addition, a scoping approach allows inclusion of pregnancy-relevant primary evidence even when neonatal or child outcomes are not the primary endpoint (e.g., exposure patterns, maternal characteristics, postpartum maternal outcomes, and biologically relevant correlates), which may be important for interpreting bias and informing pragmatic clinical counseling.^17–20,31–34^

### Objectives and review questions

The primary objective of this scoping review is to map and synthesize the extent, nature, and limitations of primary human evidence describing prenatal exposure to MDMA, psilocybin, and classic hallucinogens and associated maternal, obstetric, neonatal, congenital anomaly, and neurodevelopmental outcomes.

The review is structured around the following questions:

1. What primary human evidence sources exist describing prenatal exposure to MDMA, psilocybin, or classic hallucinogens (including LSD, mescaline/peyote, and DMT/ayahuasca)?
2. What outcome domains have been reported (maternal, obstetric, neonatal, congenital anomalies, neurodevelopment), and at what follow-up horizons?
3. How have studies handled major validity threats, including polysubstance use, exposure ascertainment, dose/timing uncertainty, and outcome definition variability?
4. What evidence gaps are most critical for clinical counseling, harm reduction, perinatal pharmacovigilance, and ethically designed future research as psychedelic therapeutic use expands?^4–9^

## Methods

### Study design and reporting framework

This scoping review was conducted and reported in accordance with the Preferred Reporting Items for Systematic Reviews and Meta-Analyses Extension for Scoping Reviews (PRISMA-ScR).^31–34^ We mapped primary human evidence describing prenatal exposure to MDMA, psilocybin, and classic hallucinogens using established scoping review methodology. The a priori protocol is provided in Supplemental Material.

### Eligibility criteria

Eligibility was defined using the Population–Concept–Context (PCC) framework.^34^ We included primary human studies (cohort, case–control, cross-sectional, case series, and case reports) involving pregnant individuals with documented exposure to MDMA, psilocybin, or classic hallucinogens (specifically LSD, mescaline/peyote, and DMT/ayahuasca). Eligible outcomes included maternal, obstetric, neonatal, congenital, and neurodevelopmental findings. All settings were eligible, including community use, clinical encounters, and poison center contacts. We excluded animal or in vitro studies, reviews, editorials, and studies limited to preconception-only exposure. Consistent with scoping methodology, studies reporting exposure patterns or maternal outcomes were included if prenatal exposure was explicitly described.^31–34^ Conference abstracts were included only when they contained extractable primary pregnancy outcome data and no corresponding full-text publication could be identified.

### Information sources and search strategy

We searched MEDLINE, Embase, PsycINFO, CINAHL, and the Cochrane Library from inception to March 10, 2025. Supplementary searches included Google Scholar (screening the first 200 results per query) and forward and backward citation chasing. Search strategies combined controlled vocabulary and keywords for pregnancy-related concepts and specific substances (e.g., MDMA, psilocybin, LSD, mescaline, DMT). Full search strategies are provided in Supplemental Material.

### Study selection and data charting

Records were deduplicated and screened independently by two reviewers at the title/abstract and full-text stages. Data were charted in duplicate using a standardized form capturing bibliographic details, study design, population characteristics, exposure features (substance, timing, and ascertainment), co-exposures, and reported outcomes.^31–34^

### Assessment of key validity threats and synthesis

Consistent with scoping review methodology, a formal risk-of-bias assessment was not performed and was not used as a basis for study exclusion.^31–34^ Instead, we conducted a pragmatic assessment of key validity threats to contextualize interpretability across heterogeneous study designs, including exposure ascertainment methods, confounding by polysubstance use, outcome definition and measurement, and completeness of reporting. These considerations were summarized descriptively to support clinical interpretation rather than to generate quantitative certainty ratings. Findings were synthesized descriptively by substance and outcome domain without meta-analysis.

### Ethics

This study synthesized publicly available literature and did not require research ethics board approval.

## Results

### Study selection

The literature search identified 2478 records from bibliographic databases (MEDLINE, Embase, PsycINFO, CINAHL, and the Cochrane Library) and 12 additional records through supplementary methods, including Google Scholar exports and citation chasing. After the removal of 980 duplicates, 1510 unique records underwent title and abstract screening. Of these, 1360 records were excluded because they did not involve prenatal exposure, reported non-human or mechanistic data only, or addressed psychedelic-assisted psychotherapy without pregnancy-specific outcomes.

**Figure 1.**
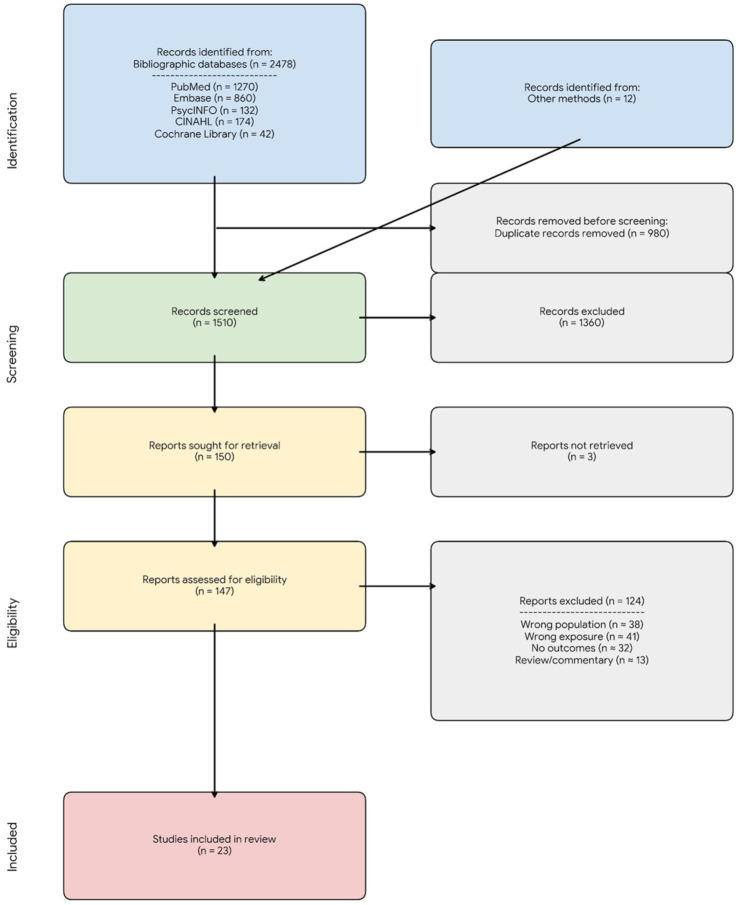
PRISMA-ScR flow diagram of study identification and selection. Records were identified through searches of bibliographic databases and other methods, including Google Scholar screening and citation chasing. After duplicate removal, titles and abstracts were screened, followed by full-text assessment for eligibility. Reasons for full-text exclusion are summarized. The final number of included studies reflects primary human evidence relevant to prenatal exposure to MDMA and classic hallucinogens. MDMA, 3,4-Methylenedioxymethamphetamine; PRISMA-ScR, Preferred Reporting Items for Systematic Reviews and Meta-Analyses Extension for Scoping Reviews.

A total of 147 full texts were assessed for eligibility. Of these, 124 were excluded due to absence of prenatal exposure, exposure limited to the preconception period, lack of extractable primary human data, or outcomes reflecting acute intoxication unrelated to pregnancy. Ultimately, 23 primary human evidence sources met the inclusion criteria and were included in this scoping review. The study selection process is summarized in the PRISMA-ScR flow diagram (Figure 1).^33^

### Characteristics of included sources

Twenty-three primary human evidence sources were included (Table 1). Evidence was concentrated in MDMA/ecstasy (*n* = 11) and LSD/lysergide (*n* = 11), with a single case report identified for mescaline/peyote (*n* = 1). No eligible primary human pregnancy outcome studies were identified for psilocybin/psilocin or DMT/ayahuasca.

**Table 1.** Included primary human sources of evidence (*n* = 23) and mapped outcome domains.

No.	Included source (Vancouver citation no.)	Substance	Design type	Outcome domains captured
1	Gilmore (2001)^30^	Mescaline/peyote	Case report	Obstetric/perinatal outcome (case-based)
2	Haden and Woods (2020)^29^	LSD	Case series	Maternal overdose; pregnancy exposure described (case-based)
3	Cohen et al. (1968)^35^	LSD	Observational (cytogenetic)	Child/chromosomal outcomes
4	Carakushansky et al. (1969)^25^	LSD (+ cannabis)	Brief report/letter	Congenital anomalies signal (case-based)
5	Aase et al. (1970)^26^	LSD	Brief report/letter	Child outcomes (case-based)
6	Assemany et al. (1970)^27^	LSD	Case report/letter	Congenital anomaly (case-based)
7	McGlothlin et al. (1970)^21^	LSD	Observational follow-up	Pregnancy outcomes
8	Hsu et al. (1970)^36^	LSD	Case report	Chromosomal abnormality case
9	Jacobson and Berlin (1972)^22^	LSD	Observational study	Reproductive/pregnancy outcomes
10	Apple and Bennett (1974)^23^	LSD	Case report	Congenital/systemic and ocular anomalies
11	Chan et al. (1978)^24^	LSD	Case report	Congenital ocular anomalies
12	von Mandach et al. (1999)^28^	LSD (+ cannabis)	Case report	Perinatal outcome (case-based)
13	McElhatton et al. (1999)^10^	MDMA	Teratology service follow-up	Congenital anomalies/pregnancy outcomes
14	Rost van Tonningen et al. (1998)^11^	MDMA	Abstract (teratology service)	Pregnancy outcomes (limited detail)
15	Rost van Tonningen-van Driel et al. (1999)^12^	MDMA	Case series	Pregnancy outcomes and malformations
16	Singer et al. (2012)^13^	MDMA	Prospective cohort	Infant neurobehavior (early infancy)
17	Singer et al. (2012)^14^	MDMA	Prospective cohort follow-up	Infant developmental outcomes (1 year)
18	Singer et al. (2015)^15^	MDMA	Prospective cohort follow-up	Developmental outcomes (subgroup analysis)
19	Singer et al. (2016)^16^	MDMA	Prospective cohort follow-up	Motor development outcomes (to 2 years)
20	Ho et al. (2001)^17^	MDMA	Prospective observational	Maternal characteristics/pregnancy-related factors
21	Moore et al. (2010)^18^	MDMA	Prospective cohort	Exposure patterns (reduction/cessation during pregnancy)
22	Turner et al. (2014)^19^	MDMA	Cohort analysis	Postpartum maternal mental health
23	Parrott et al. (2014)^20^	MDMA	Cohort analysis	Pregnancy-related biological correlates

LSD, lysergic acid diethylamide; MDMA, 3,4-methylenedioxymethamphetamine.

The included literature spanned publication years from 1968 to 2020. LSD-related evidence consisted largely of older observational reports, letters, and case reports published between the late 1960s and late 1970s, with one more recent case series. In contrast, most MDMA-related evidence was published from the late 1990s onward. Study designs were heterogeneous and included prospective observational and cohort studies (predominantly MDMA-focused), teratology information service follow-up reports and case series, and predominantly case-based and observational reporting in the LSD literature.

Across substances, exposure ascertainment relied frequently on self-report or clinical documentation rather than biological confirmation, and polysubstance co-exposure was commonly described, limiting causal inference.^13–16,21–29^

A structured summary of how prenatal exposures were characterized across included studies, including reporting of gestational timing, route of administration, exposure pattern, and exposure verification, is presented in Table 2. Reporting of these features was variable and frequently incomplete, particularly in older case-based literature.

**Table 2.** Summary of exposure characterization across included primary human sources.

Substance	Timing reported?	Route reported?	Frequency/pattern	Exposure verification
MDMA/ecstasy	Often reported but variable; gestational timing inconsistently specified	Primarily oral	Recreational use; repeated exposure common; polysubstance co-use frequent	Predominantly self-report; limited biological confirmation
LSD/lysergide	Frequently unclear or incompletely specified	Primarily oral	Sporadic or case-based exposure; polysubstance use reported in some cases	Self-report; no consistent biological verification
Mescaline/peyote	Limited reporting	Not consistently specified	Single case-based report	Self-report
Psilocybin/psilocin; DMT/ayahuasca	No eligible primary human pregnancy outcome studies identified	—	—	—

This table summarizes how prenatal psychedelic exposures were characterized across included studies, including reporting of gestational timing, route of administration, exposure pattern, and method of exposure verification. Descriptors reflect patterns observed across studies rather than uniform reporting standards.

DMT, *N,N*-Dimethyltryptamine; LSD, lysergic acid diethylamide; MDMA, 3,4-Methylenedioxymethamphetamine.

### Evidence map by substance and outcome domain

The distribution of outcome domains across substances is summarized in Table 3.

**Table 3.** Evidence map of included primary human sources by substance and outcome domain.

Substance	Maternal/exposure patterns	Obstetric outcomes	Neonatal outcomes	Congenital anomalies	Neurodevelopment/child outcomes
MDMA/ecstasy	17–20	10–12	13–16	10–12	13–16
LSD/lysergide	22, 27, 29	21, 27, 29	23–26, 28	23–26, 35, 36	25, 26, 35, 36
Mescaline/peyote	—	30	30	—	—
Psilocybin/psilocin	No eligible primary human sources identified	—	—	—	—
DMT/ayahuasca	No eligible primary human sources identified	—	—	—	—

Cells list the included citation numbers contributing evidence to that domain.

DMT, *N,N*-Dimethyltryptamine; LSD, lysergic acid diethylamide; MDMA, 3,4-Methylenedioxymethamphetamine.

#### MDMA/ecstasy

Primary human pregnancy evidence for MDMA/ecstasy comprised teratology information service follow-up reports and case series reporting pregnancy outcomes and congenital anomalies,^10–12^ as well as prospective cohort studies reporting neonatal neurobehavioral outcomes and longer-term infant and child developmental findings.^13–16^ Cohort studies included follow-up extending to approximately 2 years of age.^13–16^ Under the broader scoping approach, additional primary pregnancy-relevant evidence described exposure patterns (including reductions in MDMA use during pregnancy), pregnancy-related maternal characteristics, postpartum maternal mental health trajectories, and biologically relevant correlates in exposed cohorts.^17–20^ Collectively, the MDMA literature represents the most developed human evidence base identified in this scoping review, but remains limited by small sample sizes, frequent polysubstance exposure, and heterogeneity in outcome measurement.^13–20^

#### LSD/lysergide

LSD-related evidence was predominantly older and case-based. Reports included letters, case reports, and observational studies describing congenital anomalies, ocular findings, and pregnancy outcomes following reported maternal LSD exposure.^21–28^ Two cytogenetic reports described chromosomal observations among children exposed in utero.^35,36^ A more recent case series described LSD overdoses and included at least one case involving early pregnancy exposure, contributing contemporary but low-inference evidence.^29^ Overall, the LSD literature was heterogeneous, largely uncontrolled, and insufficient for quantitative synthesis, but it provides historically important signals and illustrates persistent limitations in exposure ascertainment and confounding control.^21–29^

#### Mescaline/peyote

Only one primary human report describing peyote use during pregnancy was identified.^30^ This isolated case-based evidence provides very limited inference and underscores the scarcity of human pregnancy literature for mescaline-related exposures compared with MDMA and LSD.

## Discussion

### Principal findings

In this scoping review, we identified a small and methodologically heterogeneous primary human evidence base describing prenatal exposure to MDMA/ecstasy and classic hallucinogens, with no eligible primary human pregnancy outcome studies identified for psilocybin/psilocin or DMT/ayahuasca. The included evidence was concentrated in MDMA-related studies (teratology service reports and prospective cohort follow-up) and older LSD-related literature (brief reports/letters, observational reports, and case reports), and interpretability was constrained by small sample sizes, reliance on self-reported exposure, frequent polysubstance co-exposures, and inconsistent or variably reported outcome definitions, limiting inference for clinical counseling and precluding quantitative synthesis.^10–29,35,36^ A single case-based report addressed mescaline/peyote exposure during pregnancy.^30^ Collectively, these findings indicate that available pregnancy evidence for psychedelic exposures remains structurally fragmented, requiring clinicians to counsel under conditions of uncertainty rather than evidence-based quantification of risk.

### Clinical implications for obstetric and addiction medicine practice

#### Counseling following MDMA exposure

MDMA/ecstasy is the most extensively studied substance in the pregnancy psychedelic literature, yet the evidence remains limited and vulnerable to confounding. Teratology service reports and case series have described pregnancy outcomes and congenital anomaly signals following reported prenatal ecstasy exposure, but these data are not sufficient to establish causality or quantify absolute risks.^10–12^ Prospective cohort follow-up studies reported early infant neurobehavioral findings and developmental outcomes extending into early childhood, providing the most clinically informative human evidence within this review; however, frequent polysubstance use and social/clinical complexity limit attribution of observed child outcomes to MDMA exposure alone.^13–16^

For counseling, these findings support a cautious, harm reduction-informed approach: clinicians should communicate that the evidence is insufficient to determine safety, that some primary studies have reported developmental concerns, and that uncertainty is amplified by co-exposures and exposure misclassification.^13–16^ In practice, risk counseling should include assessment of co-occurring substance use, psychosocial vulnerability, and comorbid psychiatric illness, and should incorporate referral pathways for addiction medicine support when indicated. Pregnancy-specific exposure pattern studies suggest that some individuals reduce or discontinue MDMA during pregnancy, but continued tobacco and cannabis use may persist, highlighting the need for comprehensive perinatal substance-use assessment beyond the index exposure.^18^

Given the pharmacological differences between MDMA and classic serotonergic psychedelics, findings from MDMA cohorts should not be generalized to substances such as LSD or psilocybin.

### Counseling following LSD exposure

The LSD/lysergide pregnancy literature was largely older and case-based, limiting interpretability and generalizability to contemporary exposures.^21–28^ Several brief reports and case reports described congenital anomalies and ocular findings following reported prenatal exposure, while observational reporting described pregnancy outcomes among LSD-exposed pregnancies; however, these sources do not establish a consistent defect pattern or provide robust risk estimates given uncertain exposure verification and high potential for reporting bias and co-exposure.^21–27^

A more recent case series involving LSD overdoses included an early pregnancy exposure, which provides contemporary but low-inference evidence relevant to emergency and addiction medicine contexts.^29^ In counseling, clinicians should emphasize that the evidence base is sparse, largely uncontrolled, and insufficient for quantifying risk; nevertheless, it supports a precautionary approach and careful clinical follow-up when exposure is reported.

### Mescaline/peyote and other classic psychedelics

Only one primary report describing peyote use during pregnancy was identified, underscoring a substantial lack of evidence for mescaline-related exposures.^30^ Similarly, our scoping review identified no eligible primary outcome studies for DMT/ayahuasca. For psilocybin, teratology summaries emphasize that human pregnancy outcome data are insufficient to evaluate miscarriage, congenital anomalies, or pregnancy complications.^9^ In the setting of absent primary evidence, counseling should explicitly differentiate “no evidence” from “evidence of no risk,” and clinicians should communicate uncertainty while reinforcing avoidance of non-medically indicated exposures during pregnancy and encouraging individualized clinical follow-up.

### Role of teratology information services and multidisciplinary care

Given the limited primary evidence and the importance of individualized risk assessment, teratology information resources (e.g., MotherToBaby fact sheets) can support patient-facing counseling by summarizing available data and documenting the degree of uncertainty.^7–9^ When exposures occur, multidisciplinary collaboration—including obstetrics, maternal–fetal medicine (where available), addiction medicine, and mental health services—may be especially valuable in addressing co-exposures, comorbid conditions, and social determinants that shape perinatal outcomes.

### Methodological limitations of the underlying evidence base (bias and interpretability)

Several recurring validity threats limited interpretability across included studies. Exposure ascertainment frequently relied on self-report and retrospective recall, with limited biological confirmation, increasing the risk of misclassification.^13–16,21–29^ Polysubstance use and confounding were common, including continued tobacco/cannabis exposure in pregnancy cohorts, which complicates attribution of outcomes to a single substance.^18^ Outcome definitions and measurement approaches were also heterogeneous, particularly for neurodevelopmental outcomes, where differences in timing, assessment tools, and follow-up completeness can materially influence observed associations.^13–16^

Importantly, these recurring limitations do not arise randomly but reflect how pregnancy drug safety evidence is typically generated in domains where pregnant and breastfeeding individuals are routinely excluded from interventional research.^4–6^ In such contexts, evidence accrues primarily through post-market observational data, teratology information services, and case-based documentation rather than through coordinated, protocolized surveillance systems.^7–9^ For psychoactive substances, under-reporting, variable drug purity, and frequent co-exposures further shape the evidentiary landscape, reinforcing structural uncertainty rather than isolated methodological weakness.^18,21–27^

These limitations were particularly pronounced in the older LSD literature, which is dominated by case reports and brief reports and is susceptible to publication bias, uncertain dosing, variable drug purity, and incomplete reporting of co-exposures and maternal comorbidity.^21–27^ Even when observational follow-up data were available, limitations in exposure verification and outcome ascertainment constrain causal interpretation.^21,22^

Finally, the broader context of pregnancy research exclusion has likely contributed to persistent evidence gaps for psychedelic substances. International and national guidance documents have highlighted how routine exclusion of pregnant and breastfeeding individuals from clinical research leads to underpowered, fragmented evidence bases, and reliance on post-market observational evidence.^4–6^ By contrast, pregnancy safety evidence in more clinically integrated pharmacologic domains—such as antidepressants and anti-epileptic medications—has often developed through sustained post-marketing surveillance systems, dedicated pregnancy exposure registries, and standardized outcome reporting frameworks, enabling progressively more coherent risk estimation over time. The relative absence of comparable infrastructure for psychedelic substances helps situate the observed evidence scarcity as historically and structurally patterned rather than anomalous. In the psychedelic domain, this gap is particularly salient given expanding therapeutic research and clinical trial activity, including MDMA-assisted therapy trials and broader psychedelic investigation for psychiatric indications.^1–3^

### Evidence gaps and research priorities

The most striking finding of this scoping review is the absence of eligible primary human pregnancy outcome studies for psilocybin/psilocin and DMT/ayahuasca, despite broad database retrieval and supplementary searching. This gap has direct implications for clinical counseling, as demand for information may increase alongside expanding psychedelic therapeutic research and changing social norms.^1–3^

Priority research directions include:

1. Prospective pregnancy exposure registries and pharmacovigilance systems for psychedelic exposures, including standardized reporting of timing, dose proxies, route, and co-exposures, with longitudinal follow-up for obstetric and child outcomes.
2. Improved exposure verification, including linkage to toxicology where feasible, and clearer characterization of drug purity and co-ingestants in non-medical exposures.
3. Standardized outcome measurement, particularly for neurodevelopment, using validated instruments and predefined follow-up horizons to enhance comparability across studies.^13–16^
4. Ethically designed inclusion strategies: Where direct inclusion in clinical trials is not feasible, trial protocols should incorporate standardized pregnancy monitoring and follow-up of inadvertent exposures to reduce data loss and strengthen real-world safety evidence, consistent with broader guidance on the ethical inclusion of pregnant individuals in research.^4–6^

### Strengths and limitations

This review was conducted using established scoping review methodology and reported in accordance with PRISMA-ScR guidance, enabling transparent and systematic mapping of a heterogeneous and historically fragmented evidence base.^31–34^ The search strategy incorporated multiple bibliographic databases from inception, along with supplementary methods (Google Scholar screening and backward and forward citation tracking), enhancing sensitivity for older literature, non-indexed sources, and case-based reports that characterize this domain.

By design, a scoping review approach prioritizes breadth and structural characterization of the literature over quantitative effect estimation.^31–34^ This was appropriate given the anticipated sparsity, methodological heterogeneity, and variability in exposure ascertainment and outcome definitions across substances. Critical appraisal was undertaken to contextualize interpretability rather than to exclude sources, allowing the review to reflect how pregnancy safety evidence in this domain is actually generated and documented.

Limitations include reliance on published literature and accessible supplementary materials, which may have resulted in under-capture of unpublished data, informal clinical reports, or non-English gray literature. As with all reviews of observational and case-based evidence, the findings are constrained by the underlying studies’ dependence on self-reported exposure, frequent polysubstance co-use, and historically inconsistent documentation practices.^31–34^ Consequently, conclusions appropriately emphasize uncertainty, structural evidence gaps, and the need for strengthened surveillance rather than causal inference or definitive risk quantification.

## Conclusion

Primary human evidence on prenatal psychedelic exposure remains limited and unevenly distributed across substances, with most available data derived from MDMA and older LSD reports.^10–29,35,36^ The available literature is structurally fragmented, shaped by the routine exclusion of pregnant individuals from interventional research and reliance on observational, case-based, and teratology service-derived evidence. In the absence of robust and standardized outcome data, clinicians should counsel patients with explicit acknowledgment of uncertainty, assess for polysubstance use and relevant comorbid conditions, and consider referral to teratology information services to support informed, shared decision-making.^7–9^

As therapeutic psychedelic research and non-medical exposure contexts continue to evolve, the development of structured perinatal pharmacovigilance systems, standardized pregnancy exposure registries, and ethically designed evidence-generation strategies is essential to improve risk characterization and inform clinical care.^1–6^ Until such infrastructure matures, counseling must proceed under conditions of acknowledged uncertainty, guided by harm reduction principles and careful clinical follow-up.

## Supplemental Material

sj-docx-1-taw-10.1177_20420986261436104 – Supplemental material for Psychedelic exposure in pregnancy: a scoping review to inform perinatal drug safety and clinical counseling Supplemental material, sj-docx-1-taw-10.1177_20420986261436104 for Psychedelic exposure in pregnancy: a scoping review to inform perinatal drug safety and clinical counseling by Ovie Martin Albert and Alexander Arthur in Therapeutic Advances in Drug Safety

## Declarations

**Ethics approval and consent to participate:** This study synthesized publicly available published literature and did not involve human participants, identifiable personal data, or new data collection. Therefore, research ethics board approval and participant consent to participate were not required.

**Consent for publication:** Not applicable. This manuscript does not contain individual person’s data in any form.

**Availability of data and materials:** All data generated or analyzed during this study are derived from publicly available published sources. The data charting form and extracted dataset are available from the corresponding author* on reasonable request.
